# Therapeutic Notes

**Published:** 1898-08-20

**Authors:** 


### THERAPEUTIC NOTES.

The solutions of the stronger drugs in the new British Pharmacopœia, 1898, remain unaltered, except that a slight change has been made in the quantities of the materials used so as to make the strength approximate more exactly to the 1 per cent. aimed at than was the case in the former edition. The following solutions are of the strength of 1 in 100:—Liquor arsenicalis, liquor arsenici hydrochloricus, liquor arsenici et hydrargyri iodidi (one of arsenic iodide and one of mercuric iodide in 100), liquor atropinæ sulphatis, liquor morphinæ acetatis, liquor morphinæ hydrochloridi, liquor morphinæ tartratis, liquor potassæ arsenitis, liquor potassii permanganatis, liquor sodii arsenatis, liquor strychninæ hydrochloridi, liquor trinitrini. The want of relation between weights and measures, however, is to be noted. In a gallon there are 70,000 grains, or 76,800 minims of water. By a simple calculation it is seen then that 100 grains of water equals 109·7143 minims, and this is so nearly 110 minims that throughout the Pharmacopœia this figure is taken as expressing the number of minims which equals 100 grains. Thus it happens that in all these solutions in which there is one gramme of the active substance in 100 cubic centigrammes, there is one grain in 110 minims. In each case, however, the proportion is to be regarded as being 1 in 100, which to all intents and purposes it is, but the directions for preparing the solutions are altered so as to make them work out more accurately.

### Cerumen in the Ear.

The utility of peroxide of hydrogen as a cleansing agent in septic conditions of the middle ear, with perforation of the drum, is well known. Dr. Waggett says that the same drug acts much more quickly than the usual bicarbonate of soda solutions in softening and disintegrating plugs of cerumen and masses of desquamated epithelium. This is a matter of some importance to the patient, for where patients are sent away with instructions to apply instillations of a solvent character, considerable pain is sometimes produced by the swelling of the sodden plug. With the peroxide solution of the strength of 20 volumes, it has been possible to remove the plug at one visit, in cases where this could only have been accomplished with difficulty by other means.

### The Inunction of Mercury.

Unna recommends that where the inunction of mercury is required it should be effected by the use of mercurial soap, rather than by the old-fashioned blue ointment. He advises potash soap, to which some benzoated lard and mercury have been added. It is much more cleanly in action than an ointment, and the mercury is said to be more quickly absorbed.

